# VAMPS: a website for visualization and analysis of microbial population structures

**DOI:** 10.1186/1471-2105-15-41

**Published:** 2014-02-05

**Authors:** Susan M Huse, David B Mark Welch, Andy Voorhis, Anna Shipunova, Hilary G Morrison, A Murat Eren, Mitchell L Sogin

**Affiliations:** 1 Department of Pathology and Laboratory Medicine, Brown University, 70 Ship Street, Campus Box G-E5, Providence, RI 02912, USA; 2 Josephine Bay Paul Center, Marine Biological Laboratory, Woods Hole, MA, USA

**Keywords:** Microbiome, Microbial ecology, Microbial diversity, Data visualization, Website, Bacteria, SSU rRNA, Next-generation sequencing

## Abstract

**Background:**

The advent of next-generation DNA sequencing platforms has revolutionized molecular microbial ecology by making the detailed analysis of complex communities over time and space a tractable research pursuit for small research groups. However, the ability to generate 10^5^–10^8^ reads with relative ease brings with it many downstream complications. Beyond the computational resources and skills needed to process and analyze data, it is difficult to compare datasets in an intuitive and interactive manner that leads to hypothesis generation and testing.

**Results:**

We developed the free web service VAMPS (Visualization and Analysis of Microbial Population Structures, http://vamps.mbl.edu) to address these challenges and to facilitate research by individuals or collaborating groups working on projects with large-scale sequencing data. Users can upload marker gene sequences and associated metadata; reads are quality filtered and assigned to both taxonomic structures and to taxonomy-independent clusters. A simple point-and-click interface allows users to select for analysis any combination of their own or their collaborators’ private data and data from public projects, filter these by their choice of taxonomic and/or abundance criteria, and then explore these data using a wide range of analytic methods and visualizations. Each result is extensively hyperlinked to other analysis and visualization options, promoting data exploration and leading to a greater understanding of data relationships.

**Conclusions:**

VAMPS allows researchers using marker gene sequence data to analyze the diversity of microbial communities and the relationships between communities, to explore these analyses in an intuitive visual context, and to download data, results, and images for publication. VAMPS obviates the need for individual research groups to make the considerable investment in computational infrastructure and bioinformatic support otherwise necessary to process, analyze, and interpret massive amounts of next-generation sequence data. Any web-capable device can be used to upload, process, explore, and extract data and results from VAMPS. VAMPS encourages researchers to share sequence and metadata, and fosters collaboration between researchers of disparate biomes who recognize common patterns in shared data.

## Background

The investigation of microbial communities has exploded in the past 10 years with the advent of next-generation DNA sequencing, uncovering an incredible diversity of microbes across different environments, from oceans to soils, from plant roots to the human body. The need to analyze marker gene datasets comprising 10^5^–10^8^ sequence reads has spawned a new generation of bioinformatics tools specifically designed for large-scale, sequence-based microbial ecology studies. Most of these tools target either quality filtering and clustering of sequences (AmpliconNoise [1], USEARCH [2]) or the assignment of taxonomy or gene function (RDP [3], SILVA [4], MG-RAST [5]). Two commonly used software packages (mothur [6] and QIIME [7]) provide a suite of programs for filtering, clustering and assigning taxonomy, with additional tools for downstream analysis. Both packages, however, require installation of software and rely on a command-line interface. Although command-line interfaces are more efficient and can be incorporated into batch processing scripts, they are not as intuitive to many users as a graphical user interface (GUI).

Ecologists and clinicians who design and conduct experiments utilizing next generation sequencing are relying more and more heavily on bioinformaticists and biostatisticians to analyze and interpret avalanches of data. So much so that the analysis of 'Big Data’ is becoming a specialized field distinct from biological interpretation. All too often, this leads to a disconnect between the researchers and their own data, relegating data visualization to the end-product of analysis, rather than an integral part of the analytical process itself [8]. We developed a free web service, *Visualization and Analysis of Microbial Population Structures* (VAMPS, http://vamps.mbl.edu), to serve as a bridge over this chasm. VAMPS offers a simple point-and-click user interface to a wide-range of visualization and analysis tools for both interactive and iterative exploration of microbial communities through comparison of marker gene data.

## Implementation

VAMPS uses PHP (v5.2.11) and JavaScript to create the website’s visual front-end and uses Apache (v2.2.25) as the web server. MySQL databases provide back-end storage of sequences, taxonomy, and user data. Large data processing requests are submitted to a cluster environment to improve overall processing speed and remove load from the primary server. Data processing and analyses employ a combination of publicly available bioinformatics tools. Quality filtering and taxonomic assignments use BioPerl scripts developed by the authors [9,10]. Operational taxonomic unit (OTU) clustering makes direct calls to source software such as UCLUST, oligotyping [11], SLP [12], and CROP [13]. Visualization and analyses utilize the R statistical environment [14] where possible, as well as QIIME and mothur.

There are no operating system, CPU, storage capacity, or memory capacity requirements: users need only a web browser and reasonable Internet connectivity. The VAMPS code is non-proprietary; however, the scale of the site and its use of multiple servers, cluster nodes, and multiple independent software packages make it infeasible for individual users to download and install locally. We welcome all users to take advantage of the computing and database storage capacity available at our website.

## Results and discussion

**Figure 1:**
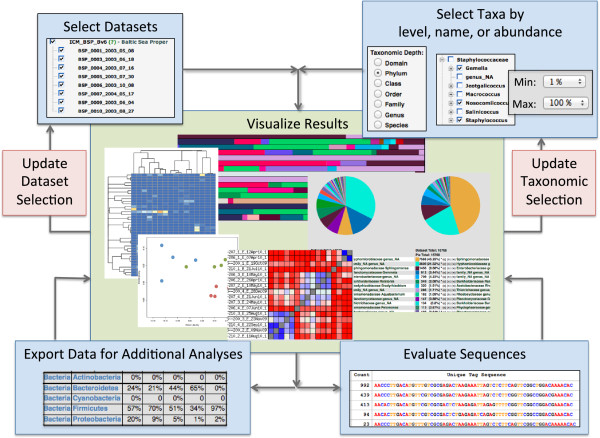
**The VAMPS website is an interactive data exploration tool that promotes iterative analysis.** Users select the datasets and taxonomic levels and classes to analyze, then visualize their microbial community structures using any of a suite of metrics including heatmaps, PCoA plots, bar graphs, pie charts and dendrograms, as well as tables of membership abundance or sequence distributions. Results from initial analyses help refine the further data selection and analytical methods. Users can download graphics, tables, data matrices, tree files, and fasta sequence files for additional analyses and for publication.

The interactive GUI encourages data exploration by enabling extensive control over sample and taxonomic selections and an intuitive path through iterative analyses and visualizations (Figure 1). This non-linear interface allows researchers to leverage their intuition and expertise in observing data patterns, leading to new insights, improved hypotheses, and a more thorough understanding of microbial communities.

VAMPS users generally start their analyses by uploading next-generation marker gene sequences, typically bacterial ribosomal RNA (rRNA) gene sequences, but also archaeal or protist rRNA or fungal ITS gene sequences. After quality filtering the uploaded sequences, VAMPS can assign taxonomic names and cluster the sequence data into OTUs using any of several commonly used algorithms (*e.g.,* oligotyping [15], reference-based clustering [16], SLP with average linkage [12], or UCLUST [2]), with or without linking to taxonomic identifiers. Alternatively, users can perform their own quality filtering, taxonomy assignments, or OTU clustering, and upload these data as input to the VAMPS analytical tools.

Although the website can be used with a public account (username “guest”), researchers who choose to upload their own data need to establish a free personal account. This account means that access to private datasets is password-protected and not session dependent. Researchers can log in and out of the website freely over the course of their research project.

VAMPS includes most common alpha and beta diversity metrics and a variety of tunable visualization approaches to explore analysis results (Figure 1). These include:

• *Heatmaps* – color-coded matrices of community similarity that can be reordered to reveal patterns among datasets and can display different beta diversity metrics above and below the diagonal;

• *Dendrograms* – tree-like diagrams clustering datasets by community similarity using one of several user-selected algorithms;

• *Principal Coordinate Analyses* – 2- and 3-D graphical representations of the relative similarity of datasets and metadata, when available;

• *Bar and Pie Charts* – graphs depicting the relative abundance of taxa or OTUs in each dataset;

• *Taxonomy and OTU Tables* – tables of absolute counts or relative abundances of sequences associated with each taxon or OTU in the selected datasets, with taxonomic names linking to NCBI, Wikipedia, and the Encyclopedia of Life and the graphing of any particular taxon or OTU abundances across the datasets;

• *Underlying sequences* – links to the sequence distributions underlying the populations, how they were taxonomically classified, and tools to search for the presence of a query sequence in the other datasets.

Users can download the analyses and images they generate on VAMPS for inclusion in publications, or they can import results from VAMPS into other software for downstream analyses. They can designate their VAMPS datasets as public or private (password-protected), and selectively share their private data with specific collaborators. Once published, datasets on VAMPS are generally made public, facilitating the data sharing required by most granting agencies and scientific journals.

**Figure 2:**
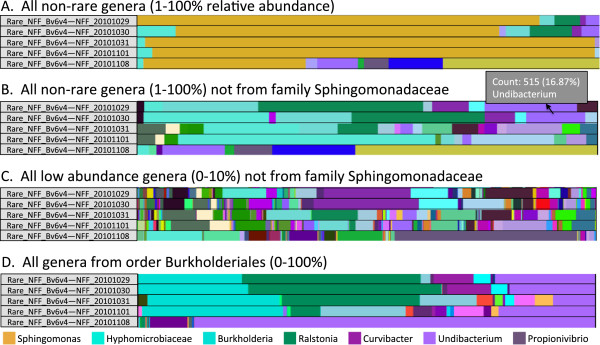
**The ability to refine the taxonomic selection facilitates exploration of both the more abundant and the less abundant taxa.** We illustrate this capability with bar charts, but these selection options are available for other analyses. **A** demonstrates an initial view of the moderately abundant (> = 1%) bacteria in the public water at a Falmouth, MA distribution point during October and November of 2011. *Sphingomonas* is the vast majority of all but one of these 5 datasets and is masking the other bacteria. To better identify the patterns within the rest of the microbial community, we can remove the Sphingomonadaceae family **(B)**. To optimize visual interpretation, the selected taxa are drawn to fill the bar graph width, but a mouse-over pop-up displays the true count of reads and relative abundance (of all taxa in the dataset, not of the subset of taxa currently displayed), the full taxonomy, and the dataset name. We have only shown the abundance and genus name here due to space considerations. We can focus in even further by looking only at taxa at less than 10% abundance in any of these datasets, still excluding Sphingomonadaceae **(C)**. Or, we can look at all genera within the order Burkholderiales which appear to be the next most abundant group of taxa after Sphingomonadaceae **(D)**.

Unique to VAMPS is its level of flexibility in taxonomy selection. Users can analyze microbial communities at any taxonomic level (domain, phylum, class, order, *etc*.), and they can also combine multiple taxonomic levels and select taxa based on abundance thresholds. For example, a user can select only taxa from a particular class, or select all taxa except a particular genus, or they can mix and match, concurrently selecting different taxonomic levels from different parts of the phylogenetic tree. As an example, a user could analyze data at the phylum level for most phyla, but at the class level for Proteobacteria and at the genus level for Bacteroidetes and Firmicutes. Users can also select taxa and OTUs based on relative abundance thresholds. This facilitates the analysis of subtle patterns of diversity obscured by dominant taxa, or conversely, the analysis of dominant or moderately abundant taxa without the potential noise of low abundance or rare taxa (Figure 2).

VAMPS also diverges from other tools by empowering users with access to the underlying sequence distributions for a selected taxon or OTU. Sequence data can be used to design further experiments, cross-check taxonomy, or query external databases. Users can interrogate the internal database for the occurrence of specific sequences in other VAMPS datasets, revealing distribution patterns of individual sequences across projects and environments. When query sequences match private datasets, users are invited to contact the anonymous owners of the private data without other aspects of the dataset being revealed, fostering new collaborations.

To facilitate comparative studies, we have loaded into VAMPS over 2,500 ready-to-use public datasets. These data have already been quality-controlled and assigned taxonomy and automatically appear in the selection window alongside the users’ own private datasets. They include data from the Human Microbiome Project (HMP) [17,18] the International Census of Marine Microbes (ICoMM) [19], the Microbial Inventory of Aquatic Long Term Ecological Research Stations (MIRADA) [20], the Census of Deep Life (CoDL) [21], and the Microbiology of the Built Environment (MoBE) [22]. Each of these projects has an entry portal with information about the projects and links to additional resources. Similar portals for other projects can be integrated into the VAMPS framework. In addition, published data from smaller projects are available for many environments including municipal water supplies, marine waters, ocean sediments, deep-sea hydrothermal vents, salt marshes, sand, and multiple biotic hosts such as humans, mice, chickens, tree leaves, and coral reefs.

**Figure 3:**
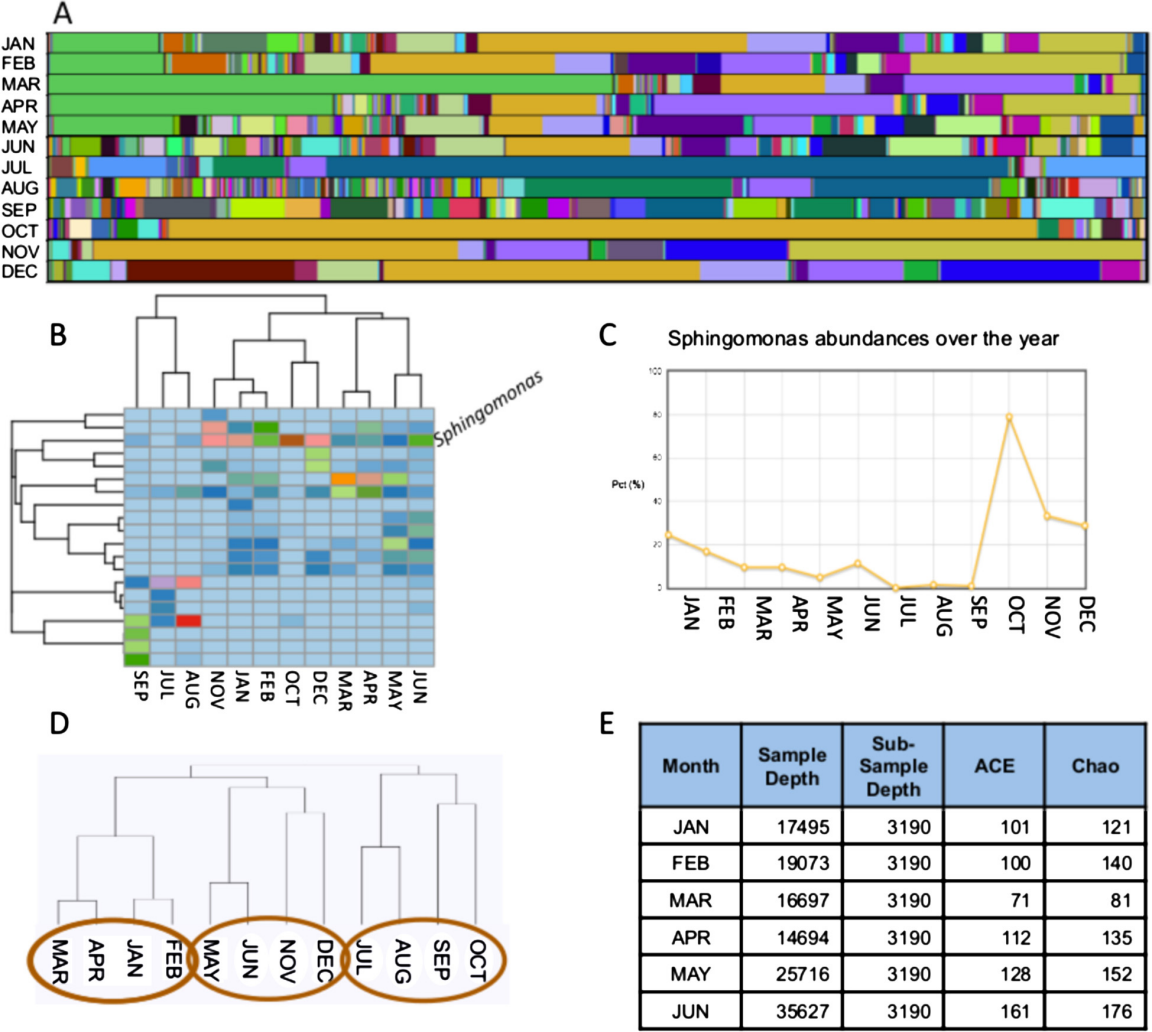
**To illustrate the use of VAMPS, we step through a simplified analysis process using water samples taken at the North Falmouth Fire Station in Falmouth, MA, project RARE_NFF_Bv6v4.** Samples were collected monthly over the course of a year (dataset names have been simplified for display). A bar chart of all taxa at the genus level **(A)** shows several consistent, abundant taxa and a large number of rare taxa particularly during May, June, August, and September. Holding the cursor over a section reveals the taxon name and abundance. For instance, the light green in the upper left is *Flavobacterium*, the ochre in the middle is *Sphingomonas*, and the lavender is *Undibacterium*. A frequency heatmap **(B)** of abundant taxa (>3% relative abundance in at least one sample) shows a dominant pattern of *Sphingomonas,* with a lower abundance in the late summer (July-September) than the rest of the year. Oddly, the months of October and November do not cluster together, nor does October cluster with September, or November with December. A graph of *Sphingomonas* abundances **(C)** shows a spike in abundance in October and November, with a gradually decreasing abundance throughout the rest of the year. If *Sphingomonas* is removed from the analysis and the data are reanalyzed in a new dendrogram, the datasets now cluster by season in three groups: late winter to early spring, late summer to fall, and the two transition times of May-June, and November-December **(D)**. Clades are neatly defined by pairs of subsequent months. This implies a possibility of two different microbial community patterns superimposed on one another that warrants further exploration. Finally, the sampling depth and alpha diversity values are exported to a table for reporting (**E**, July-December not shown).

As an illustrative vignette, Figure 3 demonstrates a simplified example analysis using water samples taken over the course of a year in Falmouth, Massachusetts, USA. We used VAMPS to explore the distribution pattern of one of the dominant taxa and its effect on the clustering of water quality samples. The example includes the use of bar charts, heatmaps, abundance graphing, dendrograms, and alpha diversity calculations.

## Conclusions

VAMPS fills a critical niche by providing ecologists and clinicians with the ability to conduct analyses that they would otherwise rely on bioinformaticians to provide. Researchers can upload and process their own data, which is maintained on the website, and, once processed, is available to use each time they log in. Its taxonomic and abundance level selection capabilities offer advantages over other programs. The underlying database includes thousands of public datasets encompassing a range of environments including the International Census of Marine Microbes and the Human Microbiome Project. These public data are accessible immediately, without download or processing, and can be analyzed separately or together with users’ private data, facilitating comparative analyses and increasing the ability to recognize important diversity patterns.

VAMPS has been instrumental in various research publications. As an example, VAMPS was used to study the microbiota of the ileal pouch of patients undergoing treatment for ulcerative colitis [23]. Analyses showed that the pouch microbiome of healthier patients evolved toward a state similar to patients with a healthy colon while the microbiome of patients prone to recurrent pouchitis tended to evolve in other directions. Additional projects have made use of the breadth of ICoMM datasets to evaluate global distributions of marine microbes [24,25], as well as the diversity of rare taxa in sand and salt marsh environments [26,27].

VAMPS is a simple-to-use website, providing universal access to microbial community marker gene data and to many visualization and analysis tools. VAMPS provides a much-needed interface for ecologists and clinicians to directly and intuitively analyze their microbial community data. The interactive nature of the website lends itself to the iterative exploratory processes so important in gaining insights into natural systems. Even for bioinformaticians well-versed with other common toolsets, the range of analyses and data visualization options and its non-linear approach makes VAMPS a valuable contribution to microbial community research.

## Availability and requirements

**Project name:** VAMPS

**Project home page:**http://vamps.mbl.edu

**Operating system(s):** Platform independent

**Programming language:** PHP, SQL, Javascript

**Other requirements:** none

**License:** GNU

**Any restrictions to use by non-academics:** none

## Competing interests

The authors declare they have no competing interests.

## Authors’ contributions

MLS conceived of the project, SMH, DMW, HGM, and MLS designed the project, evaluated software capabilities, and provided feedback on website implementation. AV, AS, and AME implemented the project. SMH, DMW, MLS and HGM wrote the manuscript. All authors read and approved the final manuscript.
